# Atypical carcinoid and large cell neuroendocrine carcinoma of the lung: a proteomic dataset from formalin-fixed archival samples

**DOI:** 10.1016/j.dib.2016.02.083

**Published:** 2016-03-09

**Authors:** Alessandro Tanca, Maria Filippa Addis, Salvatore Pisanu, Marcello Abbondio, Daniela Pagnozzi, Albino Eccher, Guido Rindi, Paolo Cossu-Rocca, Sergio Uzzau, Giuseppe Fanciulli

**Affiliations:** a Porto Conte Ricerche, Tramariglio, Alghero, Italy; b U.O. di Anatomia Patologica, Azienda Ospedaliera Universitaria Integrata di Verona, Verona, Italy; c Istituto di Anatomia Patologica, Università Cattolica del Sacro Cuore-Policlinico A. Gemelli, Rome, Italy; d U.O. di Anatomia Patologica, Azienda Sanitaria Locale di Olbia, Olbia, Italy; e Dipartimento di Scienze Biomediche, Università di Sassari, Sassari, Italy; f NET Unit, Dipartimento di Medicina Clinica e Sperimentale, Università di Sassari, AOU Sassari, Sassari, Italy

**Keywords:** Neuroendocrine tumors, Proteomics, FFPE, Archival tissues, Mass spectrometry

## Abstract

Here we present a dataset generated using formalin-fixed paraffin-embedded archival samples from two rare lung neuroendocrine tumor subtypes (namely, two atypical carcinoids, ACs, and two large-cell neuroendocrine carcinomas, LCNECs). Samples were subjected to a shotgun proteomics pipeline, comprising full-length protein extraction, SDS removal through spin columns, in solution trypsin digestion, long gradient liquid chromatography peptide separation and LTQ-Orbitrap mass spectrometry analysis. A total of 1260 and 2436 proteins were identified in the AC and LCNEC samples, respectively, with FDR <1%. MS data are available in the PeptideAtlas repository at http://www.peptideatlas.org/PASS/PASS00375.

**Specifications Table**

**Table:** 

Subject area	*Biology*
More specific subject area	*Proteomics*
Type of data	*Tables, output files, mass spectrometry files*
How data was acquired	*LTQ-Orbitrap Velos mass spectrometer (Thermo Scientific)*
Data format	*Raw, processed*
Experimental factors	*Formalin fixation, paraffin embedding, and storage performed following standard hospital procedures*
Experimental features	*High-temperature protein extraction in SDS-based reducing buffer, SDS removal using Detergent Removal Spin Columns (Pierce, Rockford, IL, USA), in solution reduction, alkylation and trypsin digestion. Peptide separation using a UltiMate 3000 RSLCnano LC system (270 min gradient).*
Data source location	*Tramariglio, Alghero (Sassari), Italy*
Data accessibility	*Data is within this article*

**Value of the data**

• Proteomic profile of atypical carcinoid.
• Largest collection of proteins identified from large-cell neuroendocrine carcinoma.
• Data might be useful for further studies aiming at the elucidation of tumor biology.
• Protein identification data can serve as benchmark for detection and verification of novel diagnostic and prognostic biomarkers of lung neuroendocrine tumors.

## 1 Data

The proteomic profiling of atypical lung carcinoid (AC) tissue is presented, together with the largest collection of proteins identified from a large-cell neuroendocrine carcinoma (LCNEC) so far. A total of 1260 and 2436 proteins were identified in the AC and LCNEC samples, respectively, with FDR <1%. Noteworthy, these data have been obtained using archival formalin-fixed paraffin-embedded (FFPE) samples, thus reinforcing the opportunity and reliability of employing such samples for clinical proteomic studies. Data files can be found at PeptideAtlas repository at http://www.peptideatlas.org/PASS/PASS00375.

## 2 Experimental design, materials and methods

Two AC and two LCNEC FFPE tissue blocks were retrieved from the repositories of the Pathology Institutes at the University Hospitals of Verona (provided by AE) and Parma (provided by GR), after approval of the respective Ethical Committees. Standard hospital procedures had been applied for fixation in formalin, embedding in paraffin, and sample storage. Hematoxylin and eosin stains had been critically reviewed and the tumors had been classified according to the lung neuroendocrine tumors World Health Organization 2004 classification. The presence of at least 80% of neoplastic tissue was verified in each FFPE tissue block, and consecutive sections were cut.

Proteins were extracted from FFPE tissues as published [1], and quantified using the EZQ Protein Quantification Kit (Molecular Probes, Eugene, OR, USA). Detergent Removal Spin Columns (Pierce, Rockford, IL, USA) were used to remove SDS, and eluates were subjected to in solution reduction, alkylation and trypsin digestion as previously described [2]. ZipTip Pipette Tips (Millipore, Billerica, MA, USA) were employed to purify peptide mixtures.

An LTQ Orbitrap Velos (Thermo Scientific, San Jose, CA, USA) interfaced with an UltiMate 3000 RSLCnano LC system (Dionex, Sunnyvale, CA, USA), set up in a data dependent MS/MS mode as described elsewhere [3], was used for MS analysis. Concentration, desalting, and separation of peptide mixtures was performed as in [2], but using a 270 min gradient from 1 to 50% eluent B (0.2% formic acid in 95% ACN) in eluent A (0.2% formic acid in 5% ACN). The gradient was as follows: 1–10% Solvent B (3 min), 10–23% B (230 min), 23–50% B (37 min). Peptide identification was performed using Proteome Discoverer (version 1.4; Thermo Scientific) as described previously [2].
