# Supplementary material for: Atypical carcinoid and large cell neuroendocrine carcinoma of the lung: a proteomic dataset from formalin-fixed archival samples
Source: Data Brief. 2016 Mar 9;7:529–31. doi: 10.1016/j.dib.2016.02.083 (PMC4796709; doi:10.1016/j.dib.2016.02.083)
Supplement: Supplementary file 1 — Supplementary material [file mmc1.doc]

Dear Editor,

I declare that there are no conflicts of interests of any of the authors concerning the submitted manuscript DIB-D-16-00111.

Maria Filippa Addis, on behalf of all authors.
